# A Newborn Screening Education Best Practices Framework: Development and Adoption

**DOI:** 10.3390/ijns5020022

**Published:** 2019-06-01

**Authors:** Adrianna Evans, Keri LeBlanc, Natasha Bonhomme, Scott M. Shone, Amy Gaviglio, Debra Freedenberg, Jeremy Penn, Carol Johnson, Beth Vogel, Siobhan M. Dolan, Aaron J. Goldenberg

**Affiliations:** 1 Genetic Alliance, 4301 Connecticut Ave NW, Suite 404, Washington, DC 20008, USA; 2 California Newborn Screening Program, Area Service Center, Rady Children’s Hospital-San Diego, 3020 Children’s Way MC 5116, San Diego, CA 92123, USA; 3 RTI International, 3040 E Cornwallis Rd, Research Triangle Park, NC 27709-2194, USA; 4 Minnesota Department of Health, 601 Robert St. N, St. Paul, MN 55155, USA; 5 Texas Department of State Health Services, Newborn Screening and Genetics Unit—MC 1918, 1100 West 49th St., Austin, TX 78756, USA; 6 The College of Education, The University of Iowa, N469 Lindquist Center, Iowa City, IA 52242, USA; 7 Stead Family Children’s Hospital, University of Iowa, 200 Hawkins Drive, Iowa City, IA 52242, USA; 8 New York State Department of Health, Wadsworth Center, 120 New Scotland Ave, Room 5020, Albany, NY 12208, USA; 9 Albert Einstein College of Medicine, Montefiore Medical Center, 1695 Eastchester Road, Room 301, Bronx, NY 10461, USA; 10 Case Western Reserve University, 10900 Euclid Ave, Cleveland, OH 44106-4976, USA

**Keywords:** newborn screening, best practices, health education, health communication, public health

## Abstract

Newborn screening is a process-based public health service. Newborn screening staff and families alike are essential to maintaining the timeliness of the screening process. Newborn screening education must be accurate and accessible. Past newborn screening conferences have highlighted gaps in best practice and evidence-based guidance on newborn screening education. Sharing successful strategies across programs mitigates the scarcity of resources by cutting costs and reducing the burden of work. These factors illustrate the need for an education framework to guide newborn screening education efforts. The Newborn Screening Education Best Practices Framework responds to these issues by outlining guidance for newborn screening education approaches. Experts in the fields of newborn screening, genetics, and bioethics as well as previous research on best practice guidelines have contributed to the development of this framework. The framework outlines a process for users to evaluate newborn screening education approaches as best practices. This framework reviews best practices using a two-step approach, looking at guiding questions, implementation of the newborn screening issue, and evaluation. The framework helps the user define the characteristics of the newborn screening issue, intended audience, and practical steps to implementation, and then decide whether or not it can be used as a best practice.

## 1. Introduction

Newborn screening (NBS) is a public health screening service that determines if babies are at risk for a number of different conditions that may be life-threatening without medical intervention [1]. In the United States (US), NBS is available to all babies born within the states and territories [1]. With four million births in the US every year, NBS requires a complex system of health professionals, laboratories, public health teams, medical staff, and processes to maintain its functionality [1]. US NBS legislation permits states and territories to select conditions for the screening panel and manage their programs separately [1]. Consequently, federal efforts can provide guidance and recommendations, but cannot mandate which conditions are included on program screening panels or standardize education nationwide [1].

Awareness is an important early step in implementing any public health service. To fully participate in public health programs, consumers must be aware of them [2,3]. Unlike other public health programs, participation in NBS does not require families’ prior knowledge of the service [1]. However, families must understand the NBS process so they can fully participate and make informed decisions about their child’s health [2,3]. By understanding the screen, families can weigh their screening options, make sure their child received the screen, understand results, and, if necessary, take appropriate action [2,3]. Subsequently, the purpose of NBS education is not solely to raise awareness about the screen. The purpose of NBS education is to help families understand the NBS process and help staff work efficiently and communicate with families. Educators require training and resources to reach the appropriate audiences.

For this reason, all staff involved in the NBS process should be well-informed about the role they play, the importance of timeliness, and how they impact the rest of the system. Providers and other staff interacting with families must support learning by communicating effectively and accurately [3]. Providers make a first impression on a family at a crucial early stage of their health journey. Providers need practical tools to support sensitive communication during the neonatal period [3]. Early interactions with pediatricians, nurses, and specialists influence family interactions with providers and the health system for years to come. Miscommunication during this time can lead to delays in treatment or negative long-term health outcomes for children and families [1].

For families, filtering through the abundance of available health information to find direction and make informed decisions can be a daunting task [3]. With media influence at an all-time high, parents are bombarded with information, expectations, opinions, and messages about what “should” or “shouldn’t” be done for the health of their child [3]. Furthermore, the time-sensitivity of NBS compounds the pressure, meaning effectiveness and timeliness in messages are critical.

NBS education approaches are highly varied across the US and other countries [1]. An education process that works in one area may not work in another, but the data each program gathers and lessons learned are valuable across different programs. In addition, NBS education is often not prioritized. Budgets for education are typically small or nonexistent, so programs need to be efficacious with funding. Sharing best practices in NBS supports efficiency in education programming and spending.

Looking at all these characteristics of NBS education, a prominent need emerges for a descriptive framework illustrating NBS education best practices. Although there are several education best practice frameworks in the broader public health realm, the distinct context of NBS requires the development of an intentional framework that accounts for the special considerations of the field. With the volume of health education strategies and programs, including individual NBS programs, advocacy groups, training and accreditation programs, and others, a framework can support standardization and streamline guidance and analysis of NBS messaging. Utilizing only effective programs across jurisdictions, programs, and hospitals will maximize funds and resources. The goal of the framework is that programs, hospitals, and other implementers will be able to thoughtfully choose an education best practice that most precisely meets the needs of their community.

## 2. Development of the NBS Education Best Practices Framework

The NBS Education Best Practices Framework was collaboratively developed with content experts in the NBS field. A workgroup comprised of US state NBS staff as well as experts in health education, ethics, policy, and public health systems all contributed to the development of this framework. The workgroup utilized an iterative process of group discussions, literature review, and writing to draft and further develop the framework presented here. The best practice concepts of broader health fields guided development of the framework [1,2,3,4,5,6,7,8,9,10,11,12,13]. With respect to other health education/communication best practices frameworks, the framework supports the unique context of NBS while also acknowledging the foundation of other work in health education. For the purpose of this framework, a best practice is defined as “a practice supported by a rigorous process of peer review and evaluation indicating effectiveness in improving health outcomes, generally demonstrated through systematic reviews.” [5]. Please see Table 1 for further definitions of terms an concepts used in the framework. 

The NBS Education Best Practices Framework is primarily a prospective tool to select appropriate, effective approaches prior to implementation, but it is also useful in evaluating past practices to determine their impact or effectiveness. The framework promotes effective education in NBS by helping the user accomplish the following:

1. Identify best practices among education approaches;
2. Select a contextually appropriate NBS education approach for implementation;
3. Move through the steps of adapting a best practice aligned with the specific goals and issues in NBS education;
4. Determine if established methods and evidence support practices;
5. Evaluate practices over time; and
6. Apply guiding questions and principles for all NBS education programs.

While anyone involved in the NBS ecosystem may find the framework useful, those responsible for the design and implementation of NBS education programs will benefit most. NBS programmatic and hospital staff can apply the framework when making choices about education strategies, programs, and materials. Though originally designed for implementation in the US NBS system, the general concepts presented in this framework are transferable to NBS programs globally, due to the focus on context.

## 3. Using the NBS Education Best Practices Framework: A Two-Step Approach

**Figure 1:**
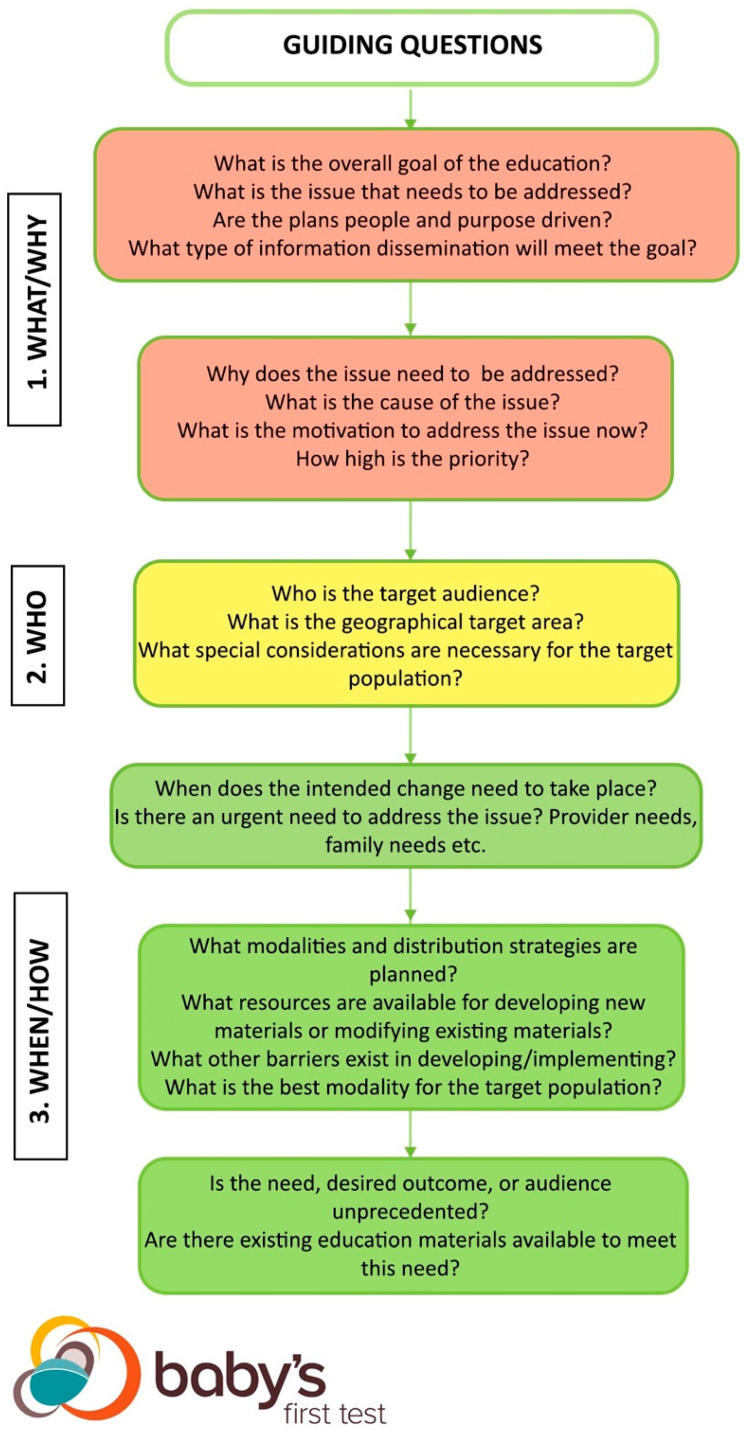
Newborn screening (NBS) Education Best Practices Guiding Questions. This figure shows the guiding questions of the NBS Education Best Practices Framework. Answering the guiding questions is the first step in using the framework. The answers to the guiding questions illustrate the core paradigms of the education approach, forming a map for users to work from in subsequent steps.

The NBS Education Best Practices Framework reviews best practices using a two-step approach. The first step examines guiding questions integral to NBS and public health education (Figure 1). The answers to the guiding questions form the core paradigms of the education approach and provide users with a list to work from when going through the subsequent step [5,7,8,9,10,11,12,13]. Once the user defines answers to the guiding questions, the user can move to the second step.

**Figure 2:**
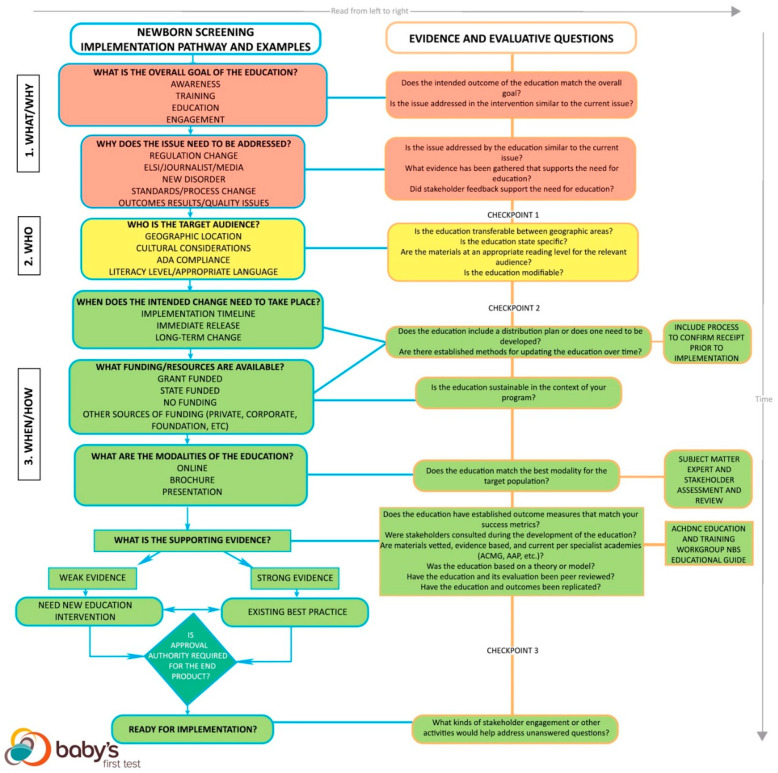
NBS Education Best Practices Flowchart. This figure shows the framework flowchart of the NBS Education Best Practices Framework. This flowchart takes the user through steps two and three of applying the framework. The flowchart highlights evaluative and implementation considerations. Users look at the answers to the guiding questions to walk through the flowchart.

The second step looks at practical implementation of an education approach, evaluation of the education approach as a best practice, and provides example considerations (Figure 2). This step identifies the context of each NBS education issue. Surrounding each education issue in NBS is a landscape of legislative, cultural, and/or community factors, so each approach must account for these variables. The evaluation process analyzes the approach itself to ascertain if it may be a best practice and if the context fits the NBS education issue.

Both the guiding questions and the flowchart have three corresponding levels: 1. What/Why, 2. Who, and 3. When/How are meant to signify checkpoints that guide users through the kinds of questions addressed at each stage of the framework. The levels correspond to stoplight colors to illustrate that users are meant to work though the framework and move towards implementation of a new or existing educational approach. At each level, users are meant to assess whether they have sufficient answers to each set of questions before moving on to the next set of questions within the framework.

### 3.1. What/Why: Goals and Factors Indicating Need

At the red “1. What/Why” level of the framework, users are asked to clearly define the overall goal of using an NBS education best practice. The changes that need to happen and why they need to happen form the foundation of a program [7,8]. By specifying the parameters of the issue and desired outcome at the beginning, the steps required to achieve the outcome emerge. The characterizations of the NBS issue should match the overall goal of the education. Different types of education are used to accomplish different goals, so it is important the approach aligns with the characteristics from the beginning.

All education arises from need. A driving factor establishes the need for education, whether it is reactive or proactive. Evidence of the need supports the rationale for the program and matches interventions to resolve problems [7,8,9,10]. In NBS, factors such as legislation, media coverage, new disorders, health outcome results, anecdotal stories from stakeholders, or quality issues can demonstrate a need for education. Evidence must substantiate the need, but evidence of a need stemming from stakeholder feedback is particularly important. As a public health service, NBS must consider the wants and needs of the stakeholders/community early and often throughout the development process.

### 3.2. Who: Target Audience

Moving downwards, the yellow “2. Who” level of the framework looks at the characteristics of the target audience for the education. An accurate understanding of the target audience is critical for intentional design and patient-centricity [8,9,11]. Audiences such as parents and families require different considerations for content and design compared to healthcare providers and vice versa. Public-facing materials need different messages, appearances, and mediums than materials intended for NBS program or hospital staff. Materials designed for the public are obligated to weigh factors such as geographic location, cultural factors such as preferences or sensitivities, translation, compliance with any legislature specific to the audience, literacy level, demographics, and access to the internet and other technologies. Some education is designed for use by a wider range of audiences, so these are modifiable for different situations. Other education interventions deal with specialized subject matter or are in specific formats that cannot be transferred to other contexts [5,11]. Charting each of these factors helps ensure the education approach is tailored to the needs of the community.

### 3.3. When/How: Implementation

Moving to the final, green “3. When/How” section of the framework, this section looks at implementation of the education approach. There are four main components to address: the implementation timeline, funding available, modalities, and whether a new education approach or an existing best practice is most appropriate. Decision-making on timeline, funding, and modality are closely integrated and may happen concurrently. Just as the audience and goal shape the decision-making process, each of these factors helps highlight the best, most practical course of action.

Depending on the driving factor of the approach, implementation timelines vary. The timeline determines the funding needed over the duration of the implementation, and, subsequently, what modalities are possible. The key to this step is identifying when the education must meet the goal. Responding to a media story requires a much shorter timeline for implementation compared to educating NBS staff about a change in policy. Similarly, the best practice should consider sustainability over a long period of time or past the availability of grant funding, if relevant. The funds available over the course of the timeline indicate what approaches or strategies are possible. If funds are not available, then plans for implementation cannot move forward. Funding must sustain the program through its duration to achieve intended goals [5,8].

The modality of the education depends on available funds and resources for distribution and development, as well as the best modality for the target audience. NBS education is often delivered through presentations, webinars, training videos, brochures, or websites, but stakeholders should direct this part of the conversation as they can identify the modalities most likely to reach their communities. Subject matter experts and stakeholder assessments should be engaged to determine which modalities are most effective for the target audience. Education approaches need to apply an appropriate modality, or there is a risk that messages will not reach the intended audience [5,8]. Some approaches can be adjusted to fit different modalities; this flexibility allows the approach to transfer between different audiences or contexts.

NBS education approaches require occasional updates. New advancements in genetics, metabolics, and precision medicine are frequently introduced. Best practices include established methods for updating the practice as advancements are made.

Established outcome measures for the evaluation of an education are another important component [5]. The outcome measures of the approach must correspond to the metrics needed to demonstrate success according to the education program or legislation.

Just as stakeholder feedback validates the goal and need, stakeholder feedback validates the plans for the approach itself. Stakeholder and community feedback is a dedicated part of a people-driven program. People-driven programs include budgeting, staff, and processes for addressing feedback throughout the duration of the program [7,8,9,10]. Stakeholders must have the opportunity to provide content expertise, address needs unique to their community, and raise additional issues. An event in the NBS field may precipitate a change to the NBS system, but stakeholder consultation informs decision-making on the mechanism of the best way to communicate the change [7,8,9,10].

The education approach must also include the planned metrics for measuring success. Education itself is qualitative, but quantitative and evidence-driven measurements reliably show progress towards education goals. For this reason, establishing metrics in the planning process is essential to demonstrate the specific changes vital to achieving the goal [8].

#### 3.3.1. Deciding on a New or Existing Education Approach

After the modality, funding, and timeline are determined, the use of an existing best practice is compared with a new program. The quality of the supporting evidence for a best practice is weighed alongside the context of the situation [5,8,12,13]. If the need, desired outcome, or audience is unprecedented in some way, then a new approach is most appropriate. If there is not strong evidence to support an education approach as a best practice, then either a new program or an appropriate best practice are acceptable. If there is strong evidence to support an approach that has passed through all the previous criteria, then that approach is an appropriate best practice. Supporting evidence should be found in academic literature, specialist academies, and evaluations of other education programs. The NBS content needed for different situations is laid out in the Educational Planning Guide created by the Education and Training Workgroup of the Advisory Committee on Heritable Disorders in Newborns and Children [14].

#### 3.3.2. Considerations for Strength of Evidence

The green “3. When/How” level of the flowchart addresses how to weigh the evidence supporting the education approach as a best practice. The strength of the evidence decides whether a new education approach or using an existing best practice is most appropriate. The user must evaluate the evidence supporting a certain practice based on several different criteria. In this framework, there are four fundamental binary questions with a scale used to judge strength. The more substantiated sources in favor of the approach, the stronger the evidence. Strength is judged on a scale of 0–4, with 0 being the weakest evidence and 4 being the strongest. Each question below answered with a “yes” gains a point [5,12,13].

1. Are materials vetted, evidence-based, and current per specialist academies or other trusted organizations?
2. Was the intervention based on a theory or model?
3. Has the intervention and its evaluation been peer reviewed?
4. Have the intervention and outcomes been replicated?

Support from specialist academies or professional organizations is an indication that experts have contributed to the education approach and that the strategies are based on methods considered valid in the field.

A model or theory also provides evidence that supports the effectiveness of the education [12,13]. Models or theories provide a methodology that forms the basis for an education approach. There are a number of public health theories that support the development of education approaches, such as the Health Belief Model, the Transtheoretical Model, Social Cognitive Theory, Social Ecological Model, and the Theory of Planned Behavior, but other theories and models may be used instead [2,3]. While they do offer some support, theories alone do not provide strong evidence, and other validating methods must be present as well.

Peer review is another way of demonstrating evidence for an education approach. Peer review of education approaches provides opportunities for experts to validate and support the methods used. Peer reviewed education approaches are described in academic journals [12,13].

Replication of the education approach with the intended outcome provides evidence for effectiveness. As the result is replicated more and more often, the evidence builds to support the fact that the education approach is meeting its goals [5,8,12,13].

#### 3.3.3. Other and Ongoing Evaluation Considerations

Finally, during the implementation process, it is important to continually evaluate and collect feedback on the use of the best practice. Even with a robust planning process, unanticipated community reactions or barriers can prevent the education from reaching its intended goals [5,8]. For this reason, every education best practice and approach must continually monitor progress towards the goal through evaluative methods and stakeholder assessment. In situations where education approaches do not achieve the intended goal, the approach requires re-evaluation [9]. Stakeholders can provide guidance and insights as to why messages are not reaching them in the intended way or having the intended impact [7,8,9,10]. Ultimately, if education approaches do not achieve the intended goal, creating a new program is the only remaining option.

## 4. Discussion: Why Education, Why Now?

With increased knowledge sharing and training on effective strategies, NBS programs are currently advancing education efforts. In the US, each state program designs their education approach for a multitude of audiences, including birth center staff, clinicians, program staff, and families [1]. In addition to the US state programs, there are national NBS education programs, such as Baby’s First Test, that create and distribute education to reach the public directly, as well as provide materials states can use and modify for their programs. The increasing complexity of the NBS system as a whole along with challenges experienced by NBS programs demonstrates the gravity of the need for public and professional NBS education. For example, Tarini discusses how the growing interest in testing and screening may lead to an increased need for healthcare providers to discuss the differences between when laboratory reports indicate an abnormal result, but a disease does not manifest [15]. Further, as public interest continues to grow in precision medicine, health equity, genetic testing, and home-based testing, NBS will garner further media attention. National scrutiny—along with the growing availability of home-based genetic tests—makes NBS education a priority now more than ever.

As these trends in public interest continue, NBS education needs to apply strategies from health education and communication. Proven principles of health education and communication provide a foundation for education that accounts for the many complicated aspects of NBS [2,3]. These principles lay the groundwork for consistent and effective messages and support standardization in awareness building throughout the NBS system [3]. Effectiveness promotes receipt and understanding of NBS messaging to the relevant audiences [3]. Together, these principles encourage coordination and alignment between various stakeholders, those implementing the health education approach, and the intended audience. Without set plans and metrics, consistency and effectiveness in education are difficult to achieve. Strategy, SMART (Specific, Measurable, Achievable, Relevant, Time-Bound) objectives, and evaluation plans are all essential components to a successful NBS education approach. To overcome this issue, the process-oriented context of NBS demands a more structured, proprietary approach to NBS education. Schiavo states: “Health communication cannot exist in a vacuum”, so NBS health education must become an integrated part of the larger NBS system [3]. The NBS Education Best Practices Framework promotes health communication standards throughout the NBS system.

## 5. Conclusions

As the field of molecular and genetic testing expands, the capabilities of NBS grow as well. Within the last several years, NBS in the US has expanded to screen infants for categories of diseases not previously screenable, such as lysosomal storage disorders and leukodystrophies. Furthermore, access to the expanded screening capabilities has not been equitable across communities within the US or other countries, so new challenges caused by language and culture may arise as screening capabilities spread. With many new possibilities on the horizon, additional challenges in education are unavoidable. The NBS Education Best Practices Framework will become integral in the coming years to guide and monitor education programs. Because context is so central to the design of the framework, it will be applicable across a wide range of programs, cultures, and audiences. Consistent use of the framework assists education efforts in creating effective NBS education approaches and, ultimately, creates better health outcomes.

## Figures and Tables

**Table 1:** Best practice concepts. This table defines concepts used in the framework.

Term	Definition	References
Best Practice	A practice supported by a rigorous process of peer review and evaluation indicating effectiveness in improving health outcomes, generally demonstrated through systematic reviews.	[5]
Stakeholder	Any group or individual who can affect or is affected by the achievement of the organization’s objectives.	[4]
Community	All who will be affected by the research results, including lay residents of a local area, practitioners, service agencies, and policymakers.	[6]
